# Aquarium Viromes: Viromes of Human-Managed Aquatic Systems

**DOI:** 10.3389/fmicb.2017.01231

**Published:** 2017-06-30

**Authors:** Yiseul Kim, William Van Bonn, Tiong G. Aw, Joan B. Rose

**Affiliations:** ^1^Department of Fisheries and Wildlife, Michigan State University, East LansingMI, United States; ^2^National Institute of Agricultural Sciences, Rural Development AdministrationWanju, South Korea; ^3^A. Watson Armour III Center for Animal Health and Welfare, John G. Shedd Aquarium, ChicagoIL, United States; ^4^Department of Global Environmental Health Sciences, School of Public Health and Tropical Medicine, Tulane University, New OrleansLA, United States

**Keywords:** antibiotic resistance genes, aquarium, metagenomics, next-generation sequencing, viromes, viruses

## Abstract

An aquarium ecosystem is home to many animal species providing conditions similar to native aquatic habitats but under highly controlled management. With a growing interest in understanding the interaction of microbiomes and resident animal health within aquarium environments, we undertook a metagenomic survey of viromes in seven aquarium systems with differing physicochemical and resident animal profiles. Our results show that a diverse array of viruses was represented in aquarium viromes, many of which were widespread in different aquarium systems (27 common viral families in all of the aquarium systems). Most viromes were dominated by DNA phages of the order *Caudovirales* as commonly found in other aquatic environments with average relative abundance greater than 64%. The composition and structure of aquarium viromes were associated with controlled system parameters, including nitrate, salinity, and temperature as well as resident animal profiles, indicating the close interaction of viromes with aquarium management practices. Furthermore, finding human associated viruses in a touch exhibit suggested that exposure of aquarium systems to human contact may lead to introduction of human cutaneous viruses into aquaria. This is consistent with the high abundance of skin microflora on the palms of healthy individuals and their detection in recreational waters, such as swimming pools. Lastly, assessment of antibiotic resistance genes (ARGs) in aquarium viromes revealed a unique signature of ARGs in different aquarium systems with trimethoprim being the most common. This is the first study to provide vital information on viromes and their unique relationships with management practices in a human-built and controlled aquarium environment.

## Introduction

Aquatic environments of public aquaria provide a home to many species of amphibians, fish, marine mammals, and reptiles for the education and enjoyment of the public, and to enhance scientific understanding of aquatic systems biology. These closed and controllable systems are intensively monitored to reduce accumulation of metabolic wastes, organic materials, and toxins through biological, chemical, and physical processes under rigorous surveillance of water quality parameters. In this way, aquaria can maintain adequate water quality and attempt to mimic the self-regulating state of native aquatic habitats. Thus, aquaria could be considered as ideal model systems to uncover organism–environment interactions.

To date, only a few studies have characterized overall population structures associated with animal species or water in aquaria. These studies have revealed that water in aquaria harbor not only a variety of animal species, but also a huge array of microorganisms. Prior to common applications of next-generation sequencing (NGS) technology, a cultivation-based study by Raja et al. (2006) conducted qualitative and quantitative examination of the microbial population in aquarium water and found large diversity but with a high abundance of *Vibrio* populations as well as other potential human pathogens. A study relying on 16S rRNA gene clone library sequencing reported 42 bacterial taxa associated with filter materials in two recirculating aquaculture systems of freshwater fish (Sugita et al., 2005). The authors observed significant differences in both bacterial density and composition between the two different systems. Another study also examined by the clone library method of 16S rRNA gene compared coral mucus-associated microbial communities between native aquatic and aquarium environments and found a significantly higher diversity of microorganisms from coral mucus in the native aquatic environment than that maintained in the closed aquaria (Kooperman et al., 2007). A more recent survey using NGS by Smith et al. (2012) suggested that aquarium water is an understudied system, composed of a total of 30 phyla, the most common being *Proteobacteria*, *Bacteroidetes*, and *Planctomycetes*. The authors also observed sequences most closely related to bacterial species that have the potential to cause diseases in fishes, humans, and other species. As the cost of NGS decreased and the interest in examining the role of the built/indoor environment in shaping patterns of microbiome increased (Kembel et al., 2012; Adams et al., 2015; Lax et al., 2015), aquariums drew special attention as ideal model systems. Thus, more recent studies using NGS found that the aquarium microbiome changes with various perturbations to the system, such as replacement of aquarium water, and with time (Van Bonn et al., 2015).

Viruses as part of the microbiome have yet to be studied in aquaria, and the number of published papers remains low and these studies are limited to viral diseases and infections of fish species important to aquaculture industry (Bernoth and Crane, 1995). Viruses are the most abundant and diverse biological entities on earth, with typical concentrations ranging from 10^9^ to 10^10^ virus-like particles (VLPs) per liter of seawater (Bergh et al., 1989; Fuhrman, 1999) and from 10^8^ to 10^9^ VLPs per gram of human feces (Kim et al., 2011). Their obligate parasitic relationships with a wide range of hosts, particularly with bacteria, affect and control the abundance and diversity of host populations and mediate horizontal gene transfer due to their ability to package DNA from one bacterial host and integrate the DNA into another bacterial host cells (Wommack and Colwell, 2000). Furthermore, phages that are believed to be the majority of the viruses in the oceans are responsible for about 10–50% of the total bacterial mortality (Suttle, 1994; Weinbauer, 2004) and release dissolved organic matter from biological cells, influencing global biogeochemical cycles (Wommack and Colwell, 2000; Weinbauer, 2004). This emphasizes the importance of examining viromes along with microbiomes in the controlled aquarium ecosystem. Parasitic relationships of viruses with a wide range of hosts, including bacteria, animals, and plants observed in the Great Lakes system (Kim et al., 2015) further highlighted the importance of understanding the role of viromes in controlling host populations and ecosystem functions.

The John G. Shedd Aquarium located in Chicago, Illinois uses Lake Michigan, processed through the city of Chicago’s municipal water system, as the source water for all of the aquarium systems, producing a wide array of freshwater and manufactured seawater environments. Each aquarium system is a unique ecosystem that is closed and controllable but mimics native aquatic habitats. Although previous studies have proved the feasibility of metagenomic approaches for exploring viromes in natural aquatic environments (Roux et al., 2012; Williamson et al., 2012; Hurwitz and Sullivan, 2013; Tseng et al., 2013; Brum et al., 2015; Kim et al., 2015, 2016), to the best of our knowledge, no studies have been published to date on viromes in an aquarium ecosystem, which is an engineered system but not wastewater (Cantalupo et al., 2011; Tamaki et al., 2012; Aw et al., 2014; O’Brien et al., 2017) or host-associated system (Minot et al., 2011; Ross et al., 2013; Lim et al., 2015). Thus, in a collaborative effort as part of Aquarium Microbiome Project^1^, we explored the relationship of viromes with exhibit environments, physiochemical parameters, and resident animals. The objectives of this study were (i) to explore taxonomic composition and structure of viromes in freshwater and seawater aquarium systems (ii) to investigate the effect of highly controlled aquarium management on virome structure as well as virus richness, (iii) to examine the impact of aquarium exposure to human contact by examining viruses potentially of human origin in touch exhibits, and (iv) to investigate the potential for antibiotic resistance genes (ARGs) encoded within the aquarium viromes.

## Materials and Methods

### Ethics Statement

Water sampling was approved by the Research Committee of the John G. Shedd Aquarium. The sampling was conducted under the guidance of aquarium husbandry and Environmental Quality Laboratory personnel.

### Sample Collection

A total of 10 60-L water samples were collected in this study. Specifically, we collected water from each of seven different aquarium exhibits, Amazon Rising (AZ), Caribbean Reef (CR), Oceanarium (OC), Stingray Touch (STA), Wild Reef (WR), and two temperature-differentiated Great Lakes systems (GLA and GLB), located within the John G. Shedd Aquarium over the course of 2 days (from April 30 to May 1, 2015). Water samples were collected by directly immersing buckets beneath the water surface. Among these exhibits, the warmer of the Great Lakes system (GLA) allows aquarium visitors to touch some resident animals whereas the colder Great Lakes system (GLB) does not. The Stingray Touch exhibit is only open to the public seasonally from late May to October and also allows aquarium visitors to touch the exhibit animals. Triplicate water samples were further collected from the Stingray Touch exhibit (STB) at the end of the season (October 9, 2015). Routine water quality surveillance included measurements of water pH, salinity, temperature, ammonia, nitrite, and nitrate. Except for the water samples collected from the Stingray Touch exhibit in October 2015, mean values of water quality measurements were calculated for the period April and May 2015. Water quality values of the Stingray Touch exhibit collected in October 2015 are mean values for the period of October 2015. The characteristics and water quality values of the seven aquarium exhibits are provided in **Table 1**. Additional information on the aquarium exhibits, including water volume and resident animal profiles is provided in Supplementary Table S1.

**Table 1:** Characteristics of aquarium exhibits.

Water type	Exhibit (abbreviation)	Temperature (°F)	Salinity (ppt)	pH	Ammonia (mg/L)	Nitrite (mg/L)	Nitrate (mg/L)	Resident animal	Plant	Disinfection system	Sample collection
Freshwater	Colder Great Lakes (GLB)	bbbbb156.6	1.0	7.20	0.02	0.010	44.8	Teleosts	Present	UV contact chamber	4/30/15
	Warmer Great Lakes (GLA)	bbbbb263.0	0.2	8.20	0.01	0.004	5.2	Amphibians, Reptiles, Teleosts	Present	None	4/30/15
	Amazon Rising (AZ)	bbbbb383.0	0.3	7.57	0.04	0.008	14.8	Elasmobranchs, Reptiles, Teleosts	Present	UV contact chamber	5/1/15

Seawater	Oceanarium (OC)	bbbbb158.6	30.5	7.96	0.00	0.149	451.3	Marine Mammals	Absent	Ozone	5/1/15
	Wild Reef (WR)	bbbbb277.1	34.0	8.15	0.01	0.011	43.6	Elasmobranchs, Teleosts	Absent	Ozone, protein fractionation	5/1/15
	Caribbean Reef (CR)	bbbbb378.9	35.5	8.01	0.01	0.014	49.8	Elasmobranchs, Sea turtle, Teleosts	Absent	Ozone, protein fractionation	5/1/15
	Stingray Touch before human contact (STA)	bbbbb479.2	34.7	8.37	0.08	0.751	10.0	None	Absent	Ozone, protein fractionation	5/1/15
	Stingray Touch after human contact (STB)	bbbbb579.3	34.1	8.02	0.00	0.020	36.7	Elasmobranchs, Teleosts	Absent	Ozone, protein fractionation	10/9/15

*Stingray Touch exhibit is open to the public seasonally from late May to October. Water samples from the Stingray Touch were collected before and after human contact. Except Stingray Touch after human contact collected on October 2015, water quality parameters, including temperature, salinity, pH, ammonia, nitrite, and nitrate are mean values for the period April and May 2015. Water quality parameters of stingray touch after human contact are mean values for the period of October 2015*.

### Generation and Sequencing of Viromes

Virome generation was performed following the procedure described by Kim et al. (2015). Briefly, viral particles in approximately 60 L of each water sample were concentrated using a hollow fiber ultrafiltration system equipped with disposable dialysis filter (30 kDa molecular weight cut-off) immediately after sample collection. Viral particles contained in the resulting concentrates (pH adjusted to 7.2) were additionally concentrated and purified by adding 10% (w/v) PEG 8000 and 0.3 M (w/v) NaCl followed by overnight incubation at 4°C and centrifugation at 11,300 × *g* for 30 min at 4°C. The viral pellet resuspended in 20 mL of phosphate-buffered saline solution (pH 7.2) was further purified by addition of chloroform (1 volume) and centrifugation at 3,000 × *g* for 30 min. The upper aqueous layer was passed through 0.22 μm sterile syringe filters. Prior to viral nucleic acid extraction, 1 mL of each purified filtrate was treated with DNase I (100 units) for 2 h at room temperature.

Extraction of viral nucleic acids was performed in duplicate using PureLink Viral RNA/DNA Mini Kit (Life Technologies, Carlsbad, CA, United States) for each of the seven samples from the first sample collection (from April 30 to May 1, 2015) to compensate for the lack of biological replicates and to minimize variation in virome preparation. No replication was prepared for extracting viral nucleic acids for each of the triplicate samples from the second sample collection (October 9, 2015). Prior to viral nucleic acid amplification, the absence of microbial cells was screened for by carrying out a PCR assay for the detection of bacterial *16S rRNA* gene product with 27F/1492R universal primers. The PCR reactions were loaded onto a 1.5% agarose gel in 1 × TAE. The extracted viral nucleic acids were reverse transcribed and amplified as described by Wang et al. (2002) to obtain a sufficient quantity of DNA and reverse transcribed RNA (cDNA) for sequencing library construction. Briefly, viral RNA was reverse transcribed with Primer A (5′-GTTTCCCAGTCACGATCNNNNNNNNN-3′) using Superscript III reverse transcriptase (Invitrogen, Carlsbad, CA, United States). Sequenase 2.0 (USB/Affymetrix, Cleveland, OH, United States) was used for second-strand cDNA synthesis and for random-primed amplification of viral DNA. Each sample was then subjected to 40 cycles of PCR amplification with Primer B (5′-GTTTCCCAGTCACGATC-3′) using AmpliTaq Gold (Life Technologies, Carlsbad, CA, United States). The PCR products were purified using PCR Clean-Up System (Promega, Madison, WI, United States) and sheared to a mean size of 200 bp (ranged from 175 to 250 bp). A total of 17 [(7 water samples × 2 extraction replicates) + 3 water samples] libraries were prepared from the size-selected fragments using the ThruPLEX^®^ DNA-seq Kit (Rubicon Genomics, Ann Arbor, MI, United States) and sequenced in a 2 bp × 100 bp paired-end (PE) format using Illumina HiSeq^TM^ 2500 Rapid Run flow cell at the Research Technology Support Facility, Michigan State University (MSU).

### Bioinformatic Analysis of Viromes

Analysis of viromes was conducted following the procedure described by Kim et al. (2015). Briefly, Illumina reads were trimmed to remove the 17-bp sequence (GTTTCCCAGTCACGATC) used for random amplification using HOMER (Heinz et al., 2010) and further excluded using FASTX-Toolkit (Goecks et al., 2010) if they (i) were shorter than 30 bp, (ii) contained ambiguous bases (Ns), or (iii) had more than 50% of bases with quality score below 30 for each data set. Based on these criteria, 7,680,158 reads were removed, leaving 927,679,526 reads (Supplementary Table S2). Illumina PE reads were then *de novo* assembled with IDBA-UD (Peng et al., 2012). The resulting contiguous reads (contigs) larger than 200 bp were blasted against the National Center for Biotechnology Information (NCBI^2^) viral reference sequence database (downloaded in June 2016) using BLASTX (Altschul et al., 1990) with an E-value cutoff of 1.0E^-5^. The BLASTX output was summarized by MEGAN (Huson et al., 2007) with parameter setting: Minimum Score = 50.0, Maximum Expected = 1.0E^-5^, Top Percent = 10.0, Minimum Support Percent = 0.0, Minimum Support = 1, and LCA Percent = 100.0. To check again the absence of cellular DNA contamination in the viromes, contigs assigned to known viruses were extracted and compared using BLASTN (Altschul et al., 1990) with an E-value cutoff of 1.0E^-30^ against the complete SILVA database (downloaded from http://ftp.arb-silva.de/release_128/Exports in June 2017). The absence of cellular DNA contamination was confirmed by the very low number of reads aligned to *16S rRNA* genes, ranging from 0 to 0.13‰ (Supplementary Table S2). Ratio of our viromes with a rDNA was lower than 0.2‰ (2 in 10,000 sequences) as defined by Roux et al. (2013), indicating that the aquarium viromes had negligible amounts of cellular contamination. Taxonomic links between phages and their cellular hosts were identified using the Virus-Host DB (Mihara et al., 2016). Furthermore, contigs were compared using BLASTX (Altschul et al., 1990) to the Comprehensive Antibiotic Resistance Database (CARD; downloaded from https://card.mcmaster.ca in November 2015), which contained 2,820 different amino acid sequences known to confer antibiotic resistance phenotypes to their hosts (McArthur et al., 2013). In order to minimize false positives, the parameter, hits with E-value < 1.0E^-30^ were selected according to Abeles et al. (2015). Abundance of ARGs in each virome was calculated by the number of reads against the contigs and normalized by the contig length and reflected by the color of scale using Microsoft Excel software.

A relative abundance for each contig was estimated by the number of reads aligned to a contig divided by the contig length using Bowtie 2 (Langmead and Salzberg, 2012) with default settings. The abundance of a viral taxonomic group was then determined by summing the abundance for each contig classified in the group. To estimate the proportion of a particular viral taxonomic group compared to the rest of the virome and to compensate for different sequencing scale, the summed relative abundance of all contigs in each virome was normalized to 100%. The summary of viral taxonomic classification was tabulated as a 17 × 90 matrix (17 viromes in rows × 90 viral families in columns). After excluding singleton taxa and combining either biological or technical replicates in each sample, this tabulet was used for constructing a presence/absence matrix with statistical seriation using PAST statistical package (Hammer et al., 2001). The relative abundance of viral families was reflected by the color of scale using Microsoft Excel software. Conocical correspondence analysis (CCA) was used to reveal relationships between viral communities and operational environmental parameters using the vegan package (Oksanen et al., 2015) in R statistical language (R Development Core Team, 2011). Variance inflation factors (VIFs; a measure for cross-correlation of explanatory variables) of environmental parameters were checked and parameters with VIFs more than 20 were eleminated (ter Braak and Šmilauer, 2002). To estimate virus richness, contig spectra from a subset of 2,500,000 quality trimmed reads from each virome was calculated with Circonspect (Angly et al., 2006) using the Minino assembler with default settings. CatchAll (Bunge et al., 2012) was employed with its default settings based on best-fit parametric based models, producing virus richness estimates. Box-plots, one-way ANOVA, and Spearman’s correlation coefficient were computed using R statistical language (R Development Core Team, 2011).

We focused on the identification of viruses potentially related to human health. Contigs associated with potential human viruses were extracted and further used as the queries in BLASTN search (Altschul et al., 1990) against the NCBI non-redundant nucleotide database (downloaded in June 2016). Reads were mapped to reference genomes of human viruses with Bowtie 2 (Langmead and Salzberg, 2012) with default settings and mapping statistics were obtained using SAMtools (Li et al., 2009) and BBMap^3^ with default settings. The quality trimmed and unassembled reads were uploaded to the Metavir server^4^ (Roux et al., 2014) to construct genome coverage plots for selected viral pathogens. A rarefaction curve was constructed within the MetaVir with clustering set at 90% identity using subsamples of 50,000 reads from each virome.

### Data Deposition

Data sets for all samples are available in the NCBI Sequence Read Archive^5^ under accession number SRP 082175.

## Results and Discussion

Seven aquarium systems were studied with the Stingray Touch system sampled before and after human contact, resulting in eight unique aquarium virome data sets. Each virome set came from systems that had different physiochemical and resident animal and plant profiles (**Table 1**) as a result of aquarium management practices for maintaining the specific environmental conditions. Three of the aquarium systems were freshwater ranging in mean temperature from 56.6 to 83.0°F, all of which had incorporated plants. Four of the systems were seawater in the mean temperature range from 58.6 to 79.3°F with no incorporated plants. Among the seven aquarium systems, OC is the only system that houses marine mammals. Two of the freshwater systems (AZ and GLB) have UV contact chambers and all of the seawater systems have either ozone or protein fractionation for disinfecting water. By generating two technical replicates for each of the seven samples (AZ, CR, GLA, GLB, OC, STA, and WR) and three biological replicates for the STB, 17 virome data colletions (14+3) resulted in 935.4 million 100-bp PE Illumina HiSeq^TM^ 2500 reads, with an average of 55.0 ± 37.8 (mean ± SD) million reads per virome (Supplementary Table S2). This helped to minimize potential bias in virome preparation and sequencing.

### Common Viruses Shared among Different Aquarium Systems

**FIGURE 1:**
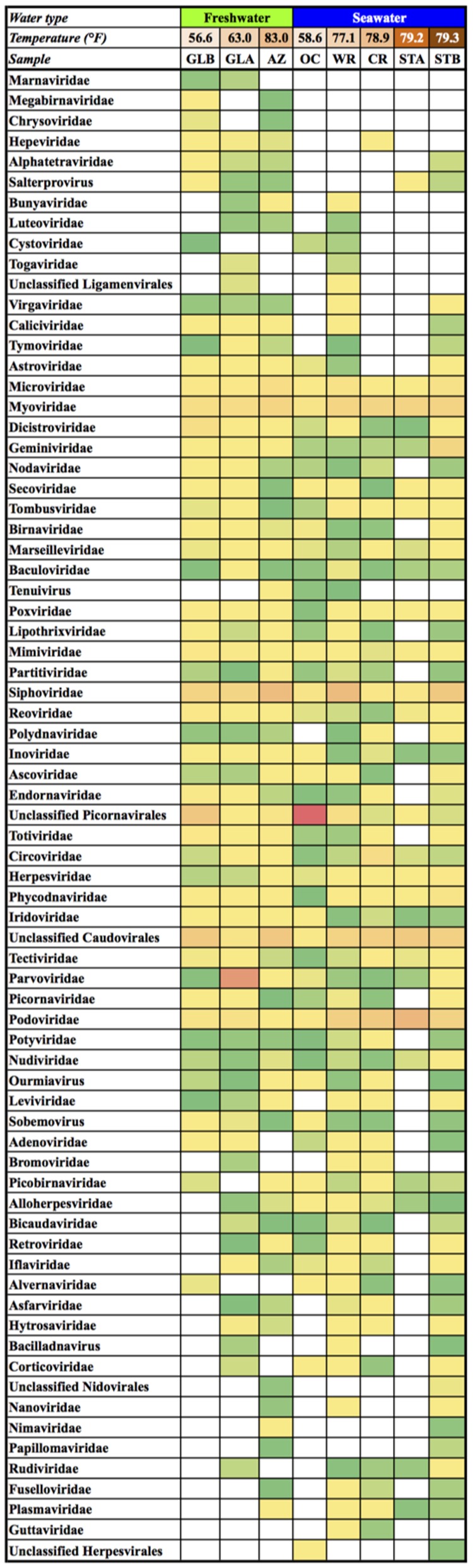
Heatmap showing the relative abundance of all identified viral families at different examined systems. The matrix has taxa as rows and samples as columns. Columns were categorized into two types of water systems and arranged from lowest to highest average water temperature under each type of water system. 17 singleton rows were removed before matrix construction. White cells indicate absence of viral families. Green is assigned to the cell with the lowest value and red with the highest value. AZ, Amazon Rising; CR, Caribbean Reef; GLA, Warmer Great Lakes; GLB, Colder Great Lakes; OC, Oceanarium; STA, Stingray Touch before human contact; STB, Stingray Touch after human contact; WR, Wild Reef.

A total of 922.7 million high quality PE reads of the 17 virome data collections were assembled, generating 928,186 contigs with an average of 54,599 ± 35,962 contigs per virome with a mean length of 938.2 ± 70.9 bp (Supplementary Table S2). Among these, 236,775 contigs (25.5% of the total number of contigs) were homologous to known viruses in the NCBI viral reference sequence database. After removing contigs lacking any taxonomic information (e.g., unclassified phages), 90 viral families were identified with 17 viral families that only occurred in a single aquarium virome (singleton taxa). As commonly used for community analysis, the single taxa were excluded from the analysis and after combining either biological or technical replicates in each sample, 73 viral families were present across the eight aquarium virome data sets (**Figure 1**). Of the 73 viral families, 23 were detected in all eight virome sets, 13 in seven virome sets, seven in six virome sets, and 10 in five virome sets. The prevalence of common viruses in the different aquarium systems observed in this study is in line with the idea that *everything is everywhere, but the environment selects* (Baas Becking, 1931; de Wit and Bouvier, 2006). This finding, however, should be interpreted with caution as some of the aquarium systems are interconnected with each other, which potentially leads to shuffling of viruses among different systems. Also, specific viral families were present only in certain aquarium viromes. For example, three viral families, *Chrysoviridae* (AZ and GLB), *Marnaviridae* (GLA and GLB), and *Megabirnaviridae* (AZ and GLB) were detected in two of the freshwater viromes but not in any of the seawater viromes.

We hypothesized that the composition of resident animals and plants as well as water salinity or tempeature play important roles in determining the distribution of viruses in the aquarium systems. We examined viromes with the lowest to highest complexity to identify factors driving distinct composition of aquarium viromes. Solely based on the number of known viral families identified by the reference sequence database, the virome of the Stingray Touch exhibit before human contact (STA), had the smallest number of known viral families (28 families) (**Figure 1**). This might be expected, as the addition of animals, had not taken place in this system at the time of sampling (animals were added starting on May 12, 2015). Water collected from the STA exhibit was municipally treated source water (from Lake Michigan) mixed with a commercial artificial sea salt product and a product containing denitrifying bacteria and ammonium chloride to encourage the growth of beneficial bacteria.

**FIGURE 2:**
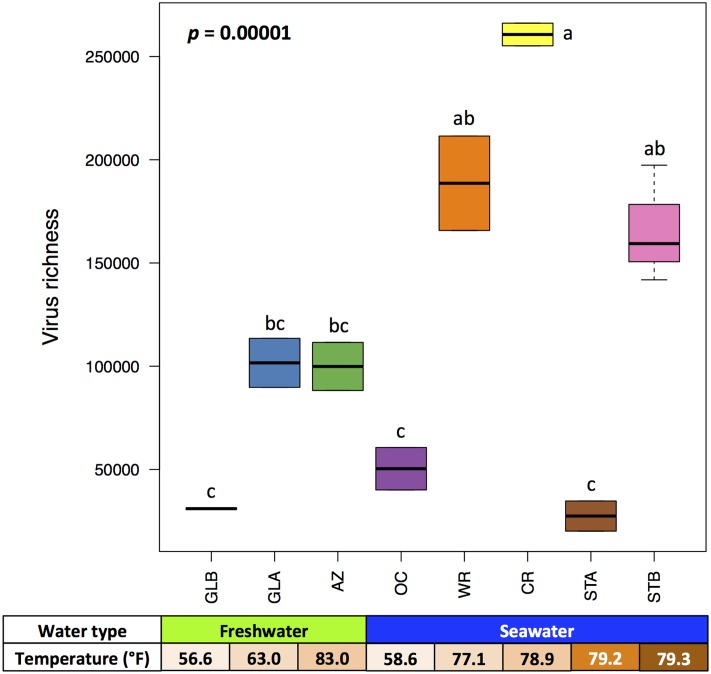
Boxplot representing virus richness of examined systems. Difference in virus richness was determined by a one-way ANOVA and *post hoc* test with Bonferroni LSD multiple comparison tests. Different letters indicate significant difference at alpha = 1%. AZ, Amazon Rising; CR, Caribbean Reef; GLA, Warmer Great Lakes; GLB, Colder Great Lakes; OC, Oceanarium; STA, Stingray Touch before human contact; STB, Stingray Touch after human contact; WR, Wild Reef.

The composition of aquarium viromes was further examined by calculating virus richness (total number of distinct viral species) from contig spectra of both known and unknown viruses due to the high percentage of unassigned viruses based on the homologous search against current NCBI viral reference sequence database. Model-based parametric richness estimates of the eight aquarium viromes using the CatchAll program ranged from 27,427.1 to 260,680.1 with an average of 108,244.6 with a significant link with the system (one-way ANOVA, *p* = 0.00001) (**Figure 2** and Supplementary Table S3). When comparing virus richness based on type of water systems, five viromes (CR, OC, STA, STB, and WR) categorized as seawater systems (141,160.1 ± 89,639.9) had higher virus richness than those (AZ, GLA, and GLB) categorized as freshwater systems (77,509.9 ± 37,529.8), but the difference was not statistically significant (*p* > 0.05) (**Supplementary Figure S1A**). When the STA virome (prior to addition of animals) was excluded from estimation of virus richness, a significant difference (*p* = 0.023) in virus richness was observed between the freshwater (77,509.9 ± 37,529.8) and seawater systems (166,434.0 ± 77,963.9) (**Supplementary Figure S1B**). This finding is consistent with a previous study, in which the highest cluster richness of viruses was observed in seawater ecosystems compared to other biomes including freshwater ecosystems (Roux et al., 2012). However, it should be noted that Roux et al. (2012) estimated cluster richness of viruses based on read clustering, while virus richness in the present study was computed based on the contig spectrum from a sequence assembly.

**FIGURE 3:**
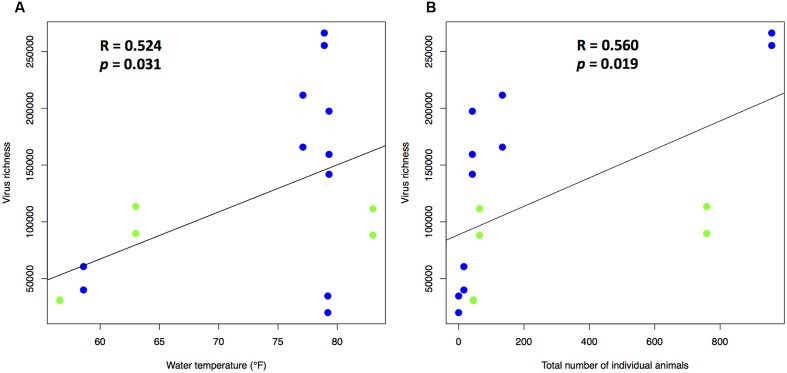
Effect of **(A)** water temperature and **(B)** total number of individual animals on virus richness (*n* = 17). Viral richness estimates were calculated using CatchAll. R was the Pearson correlation coefficient for the virus richness against the variables. Blue and green dots represent seawater and freshwater systems, respectively.

We further investigated the impact of aquarium management on virus richness in the eight viromes. Correlation analyses between virus richness and physicochemical profiles of the aquarium systems revealed that virus richness was positively correlated only with water temperature (*R* = 0.524, *p* = 0.031) (**Figure 3A**) among all the physicochemical parameters. Resident animal profiles, including number of animal types, total number of individual animals, and animal density (total number of individual animals divided by total volumes of water (gallon) in a system) were included in the correlation analyses. Significant positive correlation existed between virus richness and total number of individual animals (*R* = 0.560, *p* = 0.019) (**Figure 3B**). These findings show that water temperature and resident animal profiles, such as total number of individual animals in the aquarium systems contribute to the composition of aquarium viromes.

### High Diversity of Viruses in the Aquarium Systems

**FIGURE 4:**
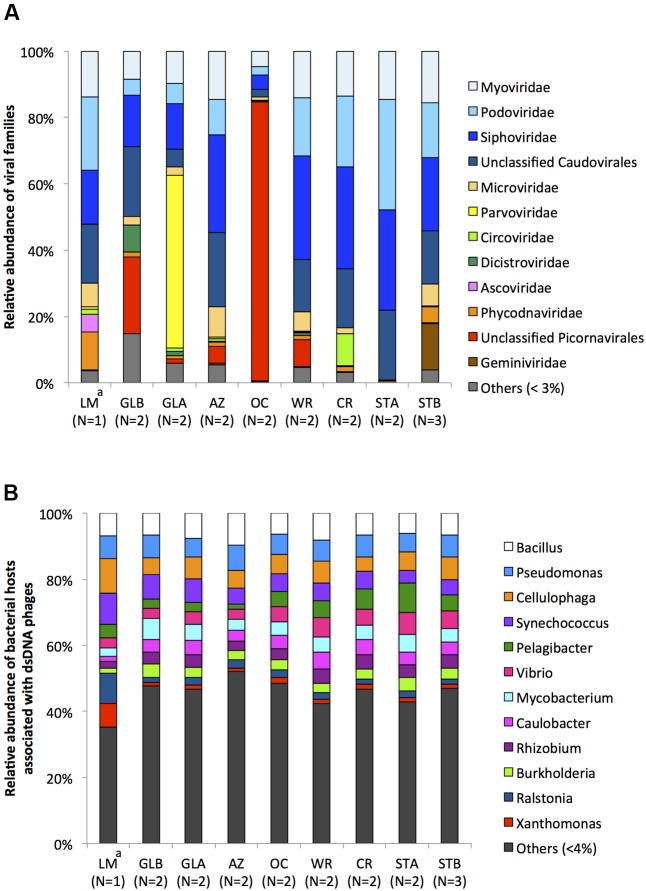
**(A)** Relative distribution of major viral families of aquarium and Lake Michigan viromes. Abundance was determined by the number of reads against the contigs divided by the contig length, and normalized to 100%. **(B)** Relative distribution of bacterial hosts associated with dsDNA phages of aquarium and Lake Michigan viromes. Abundance was represented as percentage of number of contigs in each virome. Number of replicates is given in parentheses. LM, Lake Michigan; AZ, Amazon Rising; CR, Caribbean Reef; GLA, Warmer Great Lakes; GLB, Colder Great Lakes; OC, Oceanarium; STA, Stingray Touch before human contact; STB, Stingray Touch after human contact; WR, Wild Reef. ^a^Data from Kim et al. (2015)^16^.

Prior to examining and comparing relative abundance of viral families between aquarium viromes, we included a virome of Lake Michigan (LM) from a previous study (Goecks et al., 2010) as a virome derived from a native aquatic ecosystem with limited intentional human manipulation. Relative abundance of viral families revealed that the STA virome had a less complex community composition, the vast majority (99.3%) of which was associated with double-stranded (ds) DNA phages belonging to the order of *Caudovirales* (*Myoviridae*, 14.6%; *Podoviridae*, 33.3%; *Siphoviridae*, 30.0%; and unclassified *Caudovirales*, 21.3%) (**Figure 4A**). As all of the aquarium systems use treated Lake Michigan water as their sources, all systems were expected to have similar patterns and structures of viromes with that of the STA virome in the beginning. However, shifts in the virome structure were thought to occur as a result of aquarium management practices, including establishment of physicochemical properties, such as salinity, temperature, and nutrient concentration and addition of resident animals and plants in the systems as shown in **Table 1**. Dominance of dsDNA phages was also found in the aquarium viromes (average 64.2%) except the GLA and OC viromes with low relative abundances of 35% and 13.7%, respectively. As phage populations depend directly on their bacterial hosts, contigs assigned to dsDNA phages were profiled to infer host range. These dsDNA phages were associated with 112 different bacterial hosts, 12 of which had maximum relative abundances across viromes higher than or equal to 4% (**Figure 4B**). These included contigs most similar to those infecting bacteria in genera containing plant pathogens, including *Ralstonia* (2.8%) and *Xanthomonas* (2.9%) and human pathogens, including *Bacillus* (7.2%), *Burkholderia* (3.1%), *Mycobacterium* (4.4%), *Pseudomonas* (6.5%), *Rhizobium* (3.6%), and *Vibrio* (4.5%). Together with dsDNA phages, single-stranded (ss) DNA phages, *Microviridae*, were present across aquarium viromes with low relative abundances (average 4%, ranged from 0.1 to 8.9%). This is different from other virome studies of aquatic environments where the high abundance of *Microviridae* has been reported (Angly et al., 2006; Roux et al., 2012; Kim et al., 2016). This indicates that difference in virome structure exists between native aquatic habitats and aquarium systems, which are maintained to ensure ideal living conditions for the resident animals.

In addition to phages, viruses infecting a broad range of hosts, including archaea, fungi, invertebrates, plants, protists, and vertebrates were present at different abundances in the aquarium viromes. Among the 10 major viral families whose maximum relative abundance across eight viromes greater than 3%, plant viruses in the family of *Geminiviridae* were observed with an average relative abundance of 1.6% (ranged from 0.001 to 13.8%). These were found with a high relative abundance in the STB virome (13.8%). This was an interesting finding, as this aquarium did not have plants incorporated into the ecosystem. The most likely reason is that the STB is the only outdoor exhibit and is surrounded by abundant gardens. Thus, the presence of a relatively high abundance of plant viruses may result from inputs from plant pollens that settled out of the air, possibly even airborne seeds or leaves. This suggests that we may be underestimating transport of airborne plant propagules and viruses.

Another characteristic of aquarium virome structure was the prevalence of viruses infecting insects. Among the 10 major viral families presented in **Figure 4**, three families, *Ascoviridae*, *Dicistroviridae*, and *Parvoviridae* (especially subfamily *Densovirinae*), known to infect insects, were found in all viromes (except *Ascoviridae* in the STA virome) with average relative abundances of 0.6, 1.2, and 6.0%, respectively. Although these insect viruses were present with relatively low abundances, this finding was also of interest. Two likely reasons for the appearance of insect viruses in the aquarium systems are feeder insects such as crickets and drosophilia that are cultured as food, and potentially pests such as cockroaches as pesticides are not used in the aquaria due to their potential effects on animal health. These assumptions were supported by best BLASTX matches of translated amino acid sequences of contigs to insect viruses, such as Cricket paralysis virus and *Drosophila* C virus (genome coverage plot for each virus is also shown in **Supplementary Figure S2**).

It is noteworthy that a high abundance of contigs belonging to unclassified *Picornavirales* was found in the virome of the OC exhibit, which houses marine mammals. While the average abundance of contigs associated with unclassified *Picornavirales* across eight aquarium viromes was 13.6%, the abundance of *Picornavirales* in the OC virome amounted to 84.2%. Instead of using absolute abundance of viral families, abundance of viral families was normalized to 100% (relative abundance) in this study to compensate for different sequencing scale and for between-sample comparison. As a result, *Picornavirales* appeared to be more abundant than other viral families within the OC virome. To avoid bias associated with using relative abundance and to confirm the prevalence of *Picornavirales* in the OC virome, absolute abundance of viral families was also examined. **Supplementary Figure S3** shows that the OC virome indeed had a high abundance of contigs associated with *Picornavirales*. Further examination of these contigs at the species level showed that they were closely related to viruses infecting algae (data not shown). It has been suggested that high nitrate concentration causes rapid algae growth, resulting in algal blooms in aquaria (Jackson, 2011). This may explain our observation, considering notably higher nitrate concentrations were observed in the OC exhibit compared to others (**Table 1**). However, an algal bloom was not observed in the exhibit before or after the sampling period. A location that potentially allows algae to grow in the exhibit out of visibility during routine management (e.g., storage tanks of water outside of the exhibit area and then get circulated into the exhibit) is possible and this remains to be explored. Moreover, further investigation is required to elucidate influences of increased nitrate concentration on algae growth, and in turn, enrichment of specific viral groups, especially algal viruses in the aquaria. Confirmation of the detection of contigs associated with algal viruses should also be carried out through gene-specific PCR or a phylogenetic approach.

### Response of Viromes to Highly Controlled Aquarium Management

The current reference sequence databases for virus taxonomy assignment limit our understanding of the whole virome data sets, as is commonly found in environmental studies (Hurwitz and Sullivan, 2013; Martinez et al., 2014; Winter et al., 2014; Kim et al., 2015, 2016). Nevertheless, examination of the distribution of viruses along the highly controlled physicochemical parameters of aquarium systems could provide an insight into the impact of aquarium management on viruses and thus on the health of the engineered ecosystems. Furthermore, understanding the factors that shape aquarium virome structures could potentially enhance management practices and thus resident animal health. In this study, six physicochemical parameters, including pH, salinity, temperature, and concentrations of ammonia, nitrite, and nitrate were measured on a regular basis and their mean values within a certain period of time were obtained (**Table 1**). To identify variables that explained significant directions of variance in the distribution of viruses, four parameters – nitrate, pH, salinity, and temperature – with VIFs less than 20 were selected prior to performing CCA. A one-way ANOVA using permutation tests showed that the model was statistically significant (*p* = 0.001), indicating that the four parameters were major factors influencing aquarium virome structures.

**FIGURE 5:**
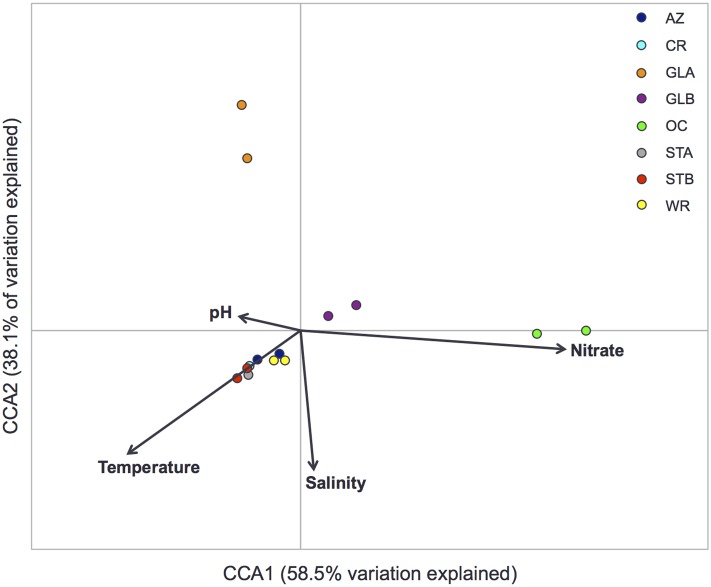
Conocical correspondence analysis (CCA) ordination plot for the first two dimensions to show the relationship between the virome structure (relative abundance of viral families) and physicochemical parameters. Correlations between physicochemical parameters and CCA axes are represented by the length and angle of arrows. AZ, Amazon Rising; CR, Caribbean Reef; GLA, Warmer Great Lakes; GLB, Colder Great Lakes; OC, Oceanarium; STA, Stingray Touch before human contact; STB, Stingray Touch after human contact; WR, Wild Reef.

The first two CCA axes (CCA1 and CCA2) explained 96.6% of the total variance in the virome structures (**Figure 5**). The ordination plot indicated that nitrate concentration and salinity of water showed a positive correlation with the first axis and a negative correlation with the second axis. In contrast, pH showed a positive correlation with the second axis and a negative correlation with the first axis. The GLB and OC viromes were primarily linked to nitrate, while the GLA virome was linked to pH. Based on the length of parameter arrow, which indicates the strength of the correlation between that parameter and community structure, nitrate appeared to be an important factor influencing virome structures. In closed aquarium systems, nitrate concentration is strictly controlled to avoid problems in the long term, such as undesirable algae growth as mentioned earlier. Nitrate originally derived from the decay of waste products of animals and plants or uneaten food is the end product of the nitrogen cycle in an aquarium and the aquarium’s biological filtration. It is typically removed from the system by water changes on a regular basis although chemical “de-nitrification” methods are sometimes employed. In a marine mammal-only system such as the OC exhibit in the present study, however, nitrate concentration tends to be higher than that typically seen in other exhibits (**Table 1**), as, unlike fishes, the marine mammals do not breathe the water. The physiologic effect of elevated nitrate concentration on fish or marine mammals, or viruses is not well-understood. A recent ocean virome study reported a significant relationship between nitrite and nitrate concentrations and virome structure, including viral morphology, populations, and protein clusters (Brum et al., 2015). Another ocean virome study by Williamson et al. (2012) revealed that virus richness was best explained by the combined effects of conductivity, nitrite and nitrate concentrations, particulate phosphorus, and pH. Direct effect of changes in nitrate concentration on virome structure remains to be further elucidated.

The importance of water salinity in virome structures has been previously reported. In a study on comparative viral metagenomics, Roux et al. (2012) showed that aquatic viromes clustered according to salinity levels despite the larger geographical distances between sample locations. This reflected genetic difference of virome structures in the different aquatic environments, including freshwater, seawater, and hypersaline. Similar results were also obtained in a freshwater virome study by Kim et al. (2015) where aquatic viromes were classified into two representative groups, freshwater virome and seawater virome, highlighting that these two different environments contain unique virome signatures. These studies agreed with our finding that water salinity was one of the influential factors on viromes structure. While the two previous studies showed a genetic similarity of virome structures between related environments according to water salinity, separation of viromes between the freshwater systems (AZ, GLA, and GLB) and the seawater systems (CR, OC, STA, STB, and WR) was not observed in our current study (**Figure 5**). This may be due to a significant impact of other physicochemical parameters, including nitrate, pH, and temperature that are highly controlled as part of aquarium management practices. Moreover, different methods used for disinfecting water in the aquarium systems may be a significant factor in shaping virome structures as virus survival is highly impacted by disinfection strategies. For example, the GLA viromes formed a separate cluster from other viromes and this could be associated with application of no disinfection strategy. Thus, further investigation, where manipulation occurs on a single parameter while others remain unchanged/controlled, is needed to observe the role of parameters of interest on viromes to be studied.

Temperature was strongly and significantly linked to community variance as shown in the CCA plot. Temperature showed a strong negative correlation with both first and second axes. While viromes of the GLA, GLB, and OC systems were primarily linked to either nitrate or pH as described earlier, temperature seemed to be a major factor linking to virome structures in the rest of the examined systems. Temperature is well-known as an important factor influencing viral dynamics in seawater ecosystems due to major effects on the abundance, distribution, and metabolism of host cells (Wommack and Colwell, 2000; Weinbauer, 2004). A recent study by Brum et al. (2015) also reported a significant relationship between temperature and virome structure in seawater environments. Overall, our finding of a significant impact of physicochemical parameters on aquarium viromes suggests that there is a response to highly controlled management of the aquatic systems.

### Viral Shedding from Human Cutaneous Microbiome via Hand Contact

Aquarium systems are generally exposed to a certain degree of human contact via various routes. The seven aquarium systems investigated in the present study were affected by varying degrees of human influences. Two systems, warmer Great Lakes (GLA) and Stingray Touch (STB), in particular, are touch exhibits and therefore had the most direct exposure to humans, as visitors are allowed to put their hands in the water and touch animals all year round and seasonally, respectively. Between the two sampling points (May 1, 2015 and October 9, 2015) for the STB exhibit, approximately 430,000 visitors touched stingrays in the exhibit. The GLA exhibit had more human exposure than the STB exhibit outside with an estimated 600,000 visitors that touch animals per year. The other five exhibits also had human impact via contact with aquarists, divers, and trainers, but to a much lower degree when compared to the GLA and STB systems.

As taxonomic profile of the aquarium viromes shown in **Figure 1** or **Figure 4** did not show significant differences between systems derived from varying degrees of human influences, we further narrowed our focus to virus species associated with humans. Interestingly, the nucleotide sequences of eight contigs from the STB virome showed best BLASTN matches to four types of human papillomaviruses (HPVs), including HPV 4, HPV 80, HPV 167, and HPV 168 (**Table 2**). HPV 80 belongs to β-HPVs and HPV 4, HPV 167, and HPV 168 γ-HPVs, all of which are cutaneous types (de Villiers et al., 2004). Read mapping to reference genomes of HPVs to verify the detection of HPVs through the BLASTN search further confirmed the presence of four types of HPVs in the STB virome (**Table 3**).

**Table 2:** Summary of the contigs with significant BLASTN hits to known human papillomaviruses obtained from the aquaria.

Sample	Contig ID	Contig length (bp)	Best hit	GenBank accession number	Nucleotide identity (%)	Query coverage (%)	E-value
STB-3	37793	623	HPV 4	X70827	99	97	0.00
STB-3	5416	2,049	HPV 4	X70827	100	99	0.00
STB-3	7432	1,674	HPV 4	X70827	99	95	0.00
STB-3	29254	731	HPV 80	Y15176	99	90	0.00
STB-3	31746	696	HPV 167	KC862318	99	98	0.00
STB-3	57985	437	HPV 167	KC862318	99	100	0.00
STB-3	63345	390	HPV 167	KC862318	100	100	0.00
STB-3	44375	558	HPV 168	KC862317	88	96	4.00E^-174^

*STB, Stingray Touch after human contact*.

**Table 3:** Summary statistics of read mapping to reference genomes of human papillomaviruses.

Reference genome	GenBank accession number	Genome length (bp)	Covered bases	Covered percent^a^
HPV 4	X70827	7,353	4,802	65.3
HPV 80	Y15176	7,427	736	9.9
HPV 167	KC862318	7,228	1,541	21.3
HPV 168	KC862317	7,204	324	4.5

^a^*Percent of reference bases covered*.

Many studies have shown that viruses represent a significant part of the cutaneous flora with a high genetic diversity on healthy skin. In particular, the commensal nature on the superficial layers of the skin in most individuals has been widely demonstrated for cutaneous β- and γ-HPVs (Antonsson et al., 2003; Chen et al., 2008; Foulongne et al., 2012). Hannigan et al. (2015) recently supported the previous findings by showing that members of the family *Papillomaviridae* were most abundant on the palm of healthy individuals. Further support to the previous findings can be found in a study where HPVs were detected in recreational water, such as swimming pool waters (La Rosa et al., 2015). As suggested by the authors, their stability in water environments is not well-understood, and the likelihood of their transmission by recreational activities and potential impact on human or animal health arising from the presence of HPVs genomes deserves to be explored further. A study by Johnson et al., (under review) conducted health assessments of all the resident animals in the system producing STA and STB viromes and found no clinically significant differences in any of health parameter values pre- and post-exposure when compared to a control non-exposed group.

Considering there was a comparable amount of exposure to humans in the GLA exhibit with that of the STB exhibit, the finding of HPVs only from the STB exhibit was interesting. One possibility is that the sequencing effort may not be sufficient for taxonomic assignment of human viruses, even though our study produced large amounts of virome sequencing data. The rarefaction curves of both GLA and STB viromes did not approach a plateau (**Supplementary Figure S4**), indicating sequencing was not exhaustive and therefore potentially made HPVs undetectable in the GLA virome. Dilution of viruses in large amounts of water could also contribute to our inability of detecting HPVs in the GLA system. In fact, the GLA (44,200 gallons) exhibit contains larger amount of water in the tanks than that in STB exhibit (18,000 gallons). Another possibility for detecting HPVs only from the STB virome is that our sampling design was based on one-time sampling. Approximately 60 L of water collected from each aquarium may not be representative of the large amounts of water, ranging from 18,000 to 3,000,000 gallons. This is further supported by the fact that detection of HPVs was observed only from one of the three biological replicates obtained from the STB exhibit (**Table 2**). Thus, more intensive sampling, such as sampling the same sites over time is needed to aid in elucidating the viral diversity associated with aquarium systems.

### ARGs in Aquarium Viromes

**FIGURE 6:**
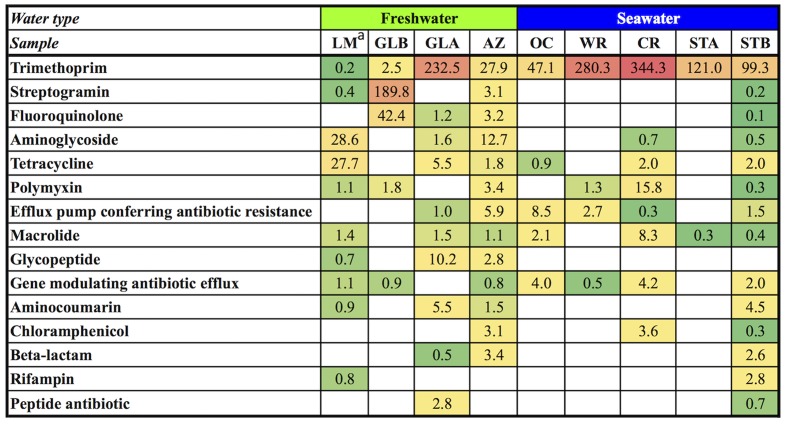
Heatmap showing the relative abundance of all identified ARGs at different examined systems. The matrix has ARGs as rows and samples as columns. Columns were categorized into two types of water systems and arranged from lowest to highest average water temperature under each type of water system. White cells indicate absence of viral families. Green is assigned to the cell with the lowest value and red with the highest value. Numbers in the heatmap cells indicate abundance of ARGs, calculated by the number of reads against the contigs and normalized by the contig length. AZ, Amazon Rising; CR, Caribbean Reef; GLA, Warmer Great Lakes; GLB, Colder Great Lakes; OC, Oceanarium; STA, Stingray Touch before human contact; STB, Stingray Touch after human contact; WR, Wild Reef. ^a^Data from Kim et al. (2015)^16^.

Aquatic environments may serve as reservoirs for the acquisition and dissemination of antibiotic resistance, because they may be impacted by the use of antibiotics to treat bacterial infections or for agricultural uses. Detection of ARGs in viromes has been reported in many diverse environments, including an aquaculture facility (Colombo et al., 2016), coprolite (Appelt et al., 2014), freshwater ponds in the Sahara desert (Fancello et al., 2013), and human skin (Hannigan et al., 2015). These studies reported that phages may harbor ARGs and alter the phenotypes of their hosts by transferring genes that confer antibiotic resistance to bacteria as summarized by Balcazar (2014). In this regard, we investigated the potential for ARGs encoded within the aquarium viromes. Viral genes conferring resistance to as many as 12 known antibiotic classes – trimethoprim, streptogramin, fluoroquinolones, aminoglycosides, tetracyclines, polymyxin, macrolides, glycopeptides, aminocoumarin, chloramphenicol, beta-lactams, and rifampin – were identified across aquarium viromes (**Figure 6**), highlighting that different aquarium systems contain unique signature of ARGs. Of these, resistance against trimethoprim was observed to be the most common (present in all nine viromes), followed by macrolides (present in seven out of nine viromes). Presence of trimethoprim resistance genes has been previously reported in other metagenomic-based environmental ARG studies, but generally with a low abundance (Ghosh et al., 2013; Nesme et al., 2014). In the aquarium, trimethoprim is sometimes used but always in combination with a sulfonamide. We did not identify genes conferring resistance to sulfonamides in the aquarium viromes. Moreover, macrolides have never been used as treatments in the studied aquarium. Aminoglycosides (4.9%), fluoroquinolones (5.2%), and tetracyclines (4.4%) were also detected across the viromes. In the studied aquarium, fluoroquinolones and tetracyclines are used infrequently, however, aminoglycosides have never been used as treatments. Similar to the taxonomic profile of aquarium viromes presented in Section “High Diversity of Viruses in the Aquarium Systems,” the STA virome showed the lowest complexity of ARG profiles with genes conferring resistance to only two known antibiotics, trimethoprim and macrolides. On the other hand, the STB virome was observed to have the highest diversity of ARGs, suggesting that the STB virome had resistance to a wider array of antibiotics with higher number of genes conferring resistance to the most kind of antibiotics than the other viromes. A significant increase in ARG diversity from STA to STB viromes suggests that aquarium management affects not only taxonomic profile but also ARG profiles within the viromes. Although ARGs have been identified in previous virome studies, including this study, potential ecological impact and public health risk of ARGs are not well-understood (Martínez et al., 2015). Moreover, a recent study by Enault et al. (2017) reported that ARG abundances in phage genomes were overestimated due to low similarities and matches to protein unrelated to antibiotic resistance. Further studies are warranted to determine ARG abundances in the environment using more conservative bioinformatics strategies and to assess impact of the presence of ARGs in viromes on public health. Lastly, detection of genes conferring resistance to unused antibiotics in the aquarium should be further investigated.

## Conclusion

An aquarium ecosystem is an ideal model system to uncover the relationships between complex viral communities and their surrounding environment. While there has been a growing interest in understanding aquarium microbiomes, studies have not yet been published on viromes within aquarium environments. Here, we investigated viromes of seven aquarium systems using metagenomic approaches for the first time. Our findings have provided several significant implications for future research and aquarium management practices. First, aquarium viromes harbor a high diversity of viruses, with prevalence of 53 common viral families shared in more than half of the aquarium systems. Most aquarium viromes were dominated by dsDNA phages of the order *Caudovirales* with average relative abundance greater than 64% along with viruses infecting many other hosts, suggesting their potential impact on aquarium systems, particularly resident animal health. Second, composition and structure of aquarium viromes respond to highly controlled aquarium ecosystem parameter values, such as nitrate, salinity, and temperature as well as resident animal profiles, emphasizing the significant impact of management practices on aquarium viromes. Third, exposure of aquarium systems to exhibit inputs, especially human contact, affects virome composition, revealing introduction of human cutaneous viruses, β- and γ-HPVs into aquarium environments. Lastly, aquarium viromes were found to harbor genes conferring resistance to 12 known antibiotics and different aquarium systems contain unique signatures of ARGs.

## Author Contributions

Conceived and designed the experiments: YK, WBV, TA, JR. Collected the samples: YK, WBV, TA. Performed the experiments: YK, TA. Analyzed the data: YK. Wrote the paper: YK, WBV, TA, JR.

## Conflict of Interest Statement

The authors declare that the research was conducted in the absence of any commercial or financial relationships that could be construed as a potential conflict of interest.

## Footnotes

1 www.aquariummicrobiomeproject.org

2 www.ncbi.nlm.nih.gov

3 http://bbmap.sourceforge.net

4 http://metavir-meb.univ-bpclermont.fr/

5 www.ncbi.nlm.nih.gov/sra

## Supplementary Material

FIGURE S1 Comparison of virus richness between freshwater and seawater aquarium systems when the STA virome was included **(A)** and excluded **(B)** as seawater system in estimation of virus richness. Difference in virus richness was determined by a one-way ANOVA test. STA, Stingray Touch before human contact.

FIGURE S2 Genome coverage plots for Cricket paralysis virus **(A)** and *Drosophila* C virus **(B)**. Number of predicted genes similar to each gene of the reference genome is given in parentheses. AZ, Amazon Rising; CR, Caribbean Reef; GLA, Warmer Great Lakes; GLB, Colder Great Lakes; OC, Oceanarium; STB, Stingray Touch after human contact; WR, Wild Reef.

FIGURE S3 Absolute abundance of contigs assigned to unclassified Picornavirales. Absolute abundance was calculated by the number of reads against the contigs and normalized by the contig length. LM, Lake Michigan; AZ, Amazon Rising; CR, Caribbean Reef; GLA, Warmer Great Lakes; GLB, Colder Great Lakes; OC, Oceanarium; STA, Stingray Touch before human contact; STB, Stingray Touch after human contact; WR, Wild Reef.

FIGURE S4 Comparison of rarefaction curves of the GLA and STB viromes. Rarefaction curves were constructed with MetaVir with clustering set at 90% identity using subsamples of 50,000 reads from each virome. GLA, Warmer Great Lakes; STB, Stingray Touch after human contact.
