# Supplementary figures and images for: Aquarium Viromes: Viromes of Human-Managed Aquatic Systems
Source: Front Microbiol. 2017 Jun 30;8:1231. doi: 10.3389/fmicb.2017.01231 (PMC5492393; doi:10.3389/fmicb.2017.01231)

**A**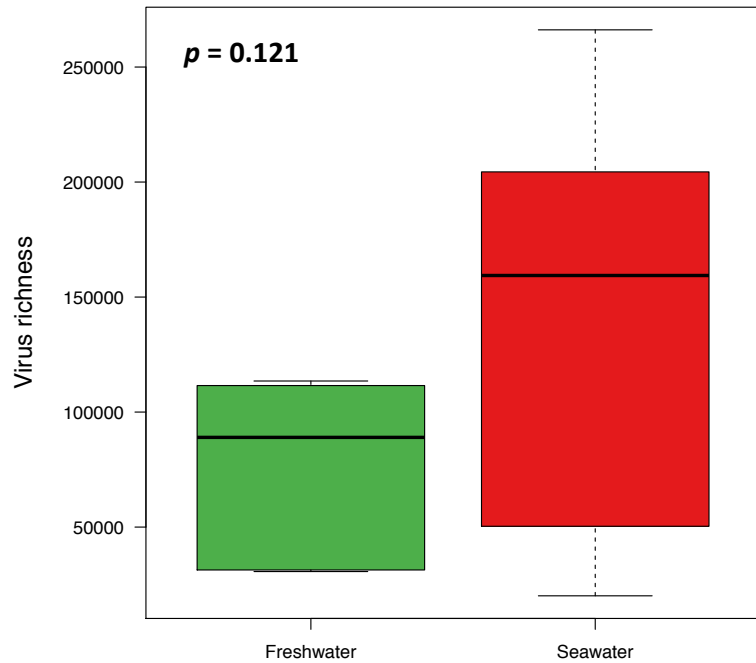**B**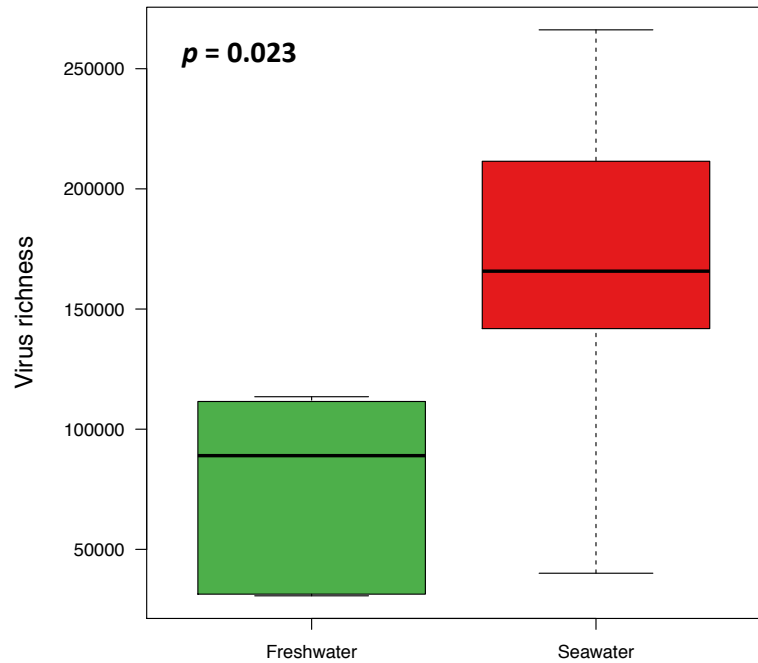

Supplement: FIGURE S1 — Comparison of virus richness between freshwater and seawater aquarium systems when the STA virome was included (A) and excluded (B) as seawater system in estimation of virus richness. Difference in virus richness was determined by a one-way ANOVA test. STA, Stingray Touch before human contact. [file Presentation_1.PDF]

A

## Cricket paralysis virus (NC003924)

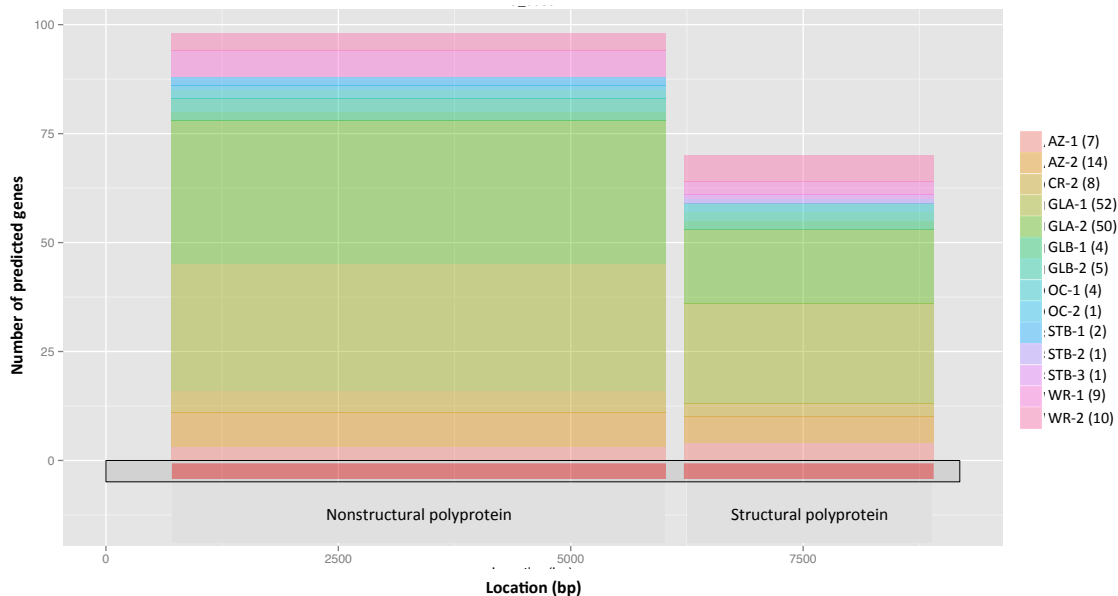

B

## Drosophila C virus (NC001834)

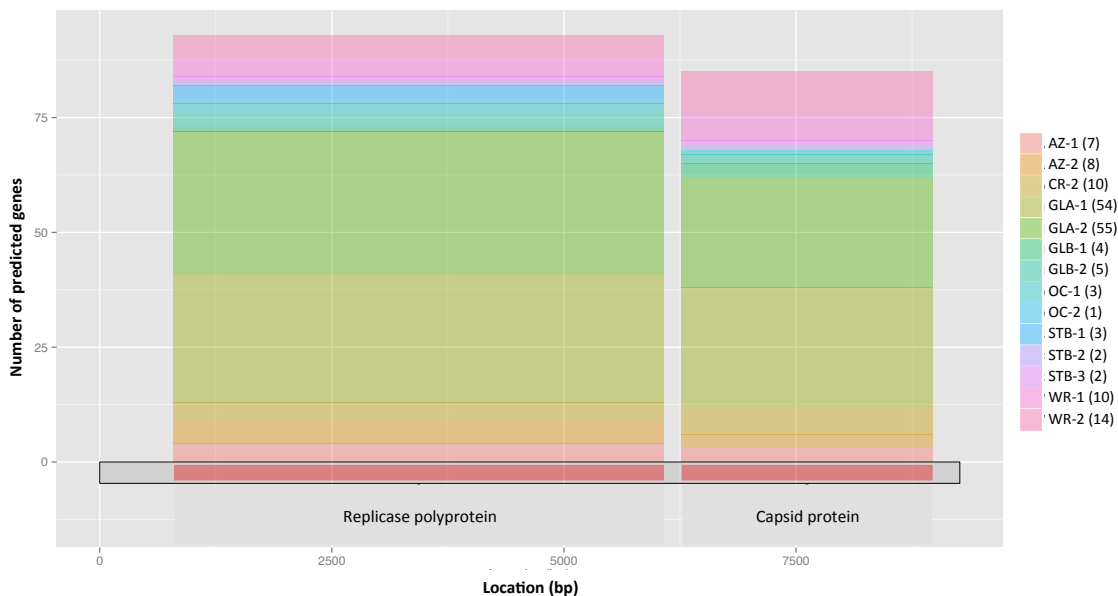

Supplement: FIGURE S2 — Genome coverage plots for Cricket paralysis virus (A) and Drosophila C virus (B). Number of predicted genes similar to each gene of the reference genome is given in parentheses. AZ, Amazon Rising; CR, Caribbean Reef; GLA, Warmer Great Lakes; GLB, Colder Great Lakes; OC, Oceanarium; STB, Stingray Touch after human contact; WR, Wild Reef. [file Presentation_2.pdf]

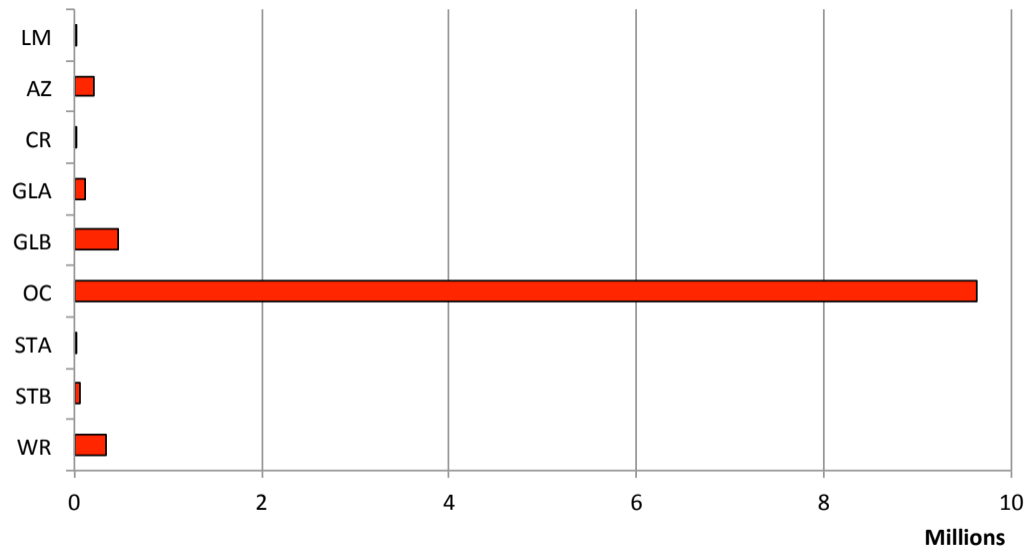

**Absolute abundance of contigs assigned to unclassified *Picornavirales***

Supplement: FIGURE S3 — Absolute abundance of contigs assigned to unclassified Picornavirales. Absolute abundance was calculated by the number of reads against the contigs and normalized by the contig length. LM, Lake Michigan; AZ, Amazon Rising; CR, Caribbean Reef; GLA, Warmer Great Lakes; GLB, Colder Great Lakes; OC, Oceanarium; STA, Stingray Touch before human contact; STB, Stingray Touch after human contact; WR, Wild Reef. [file Presentation_3.PDF]

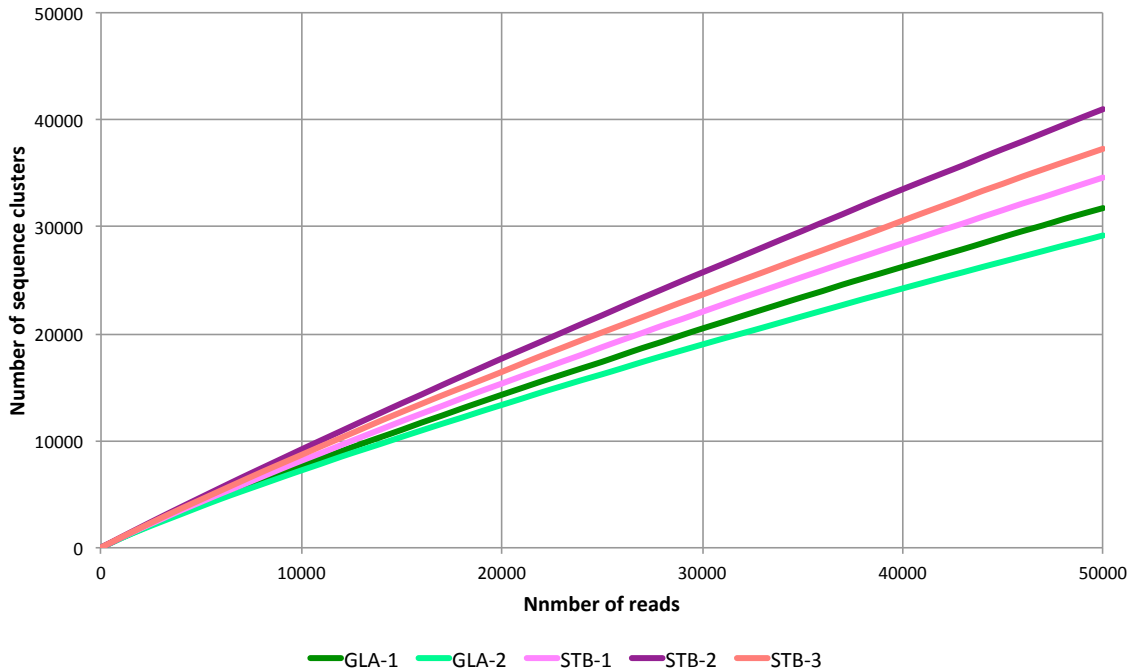

Supplement: FIGURE S4 — Comparison of rarefaction curves of the GLA and STB viromes. Rarefaction curves were constructed with MetaVir with clustering set at 90% identity using subsamples of 50,000 reads from each virome. GLA, Warmer Great Lakes; STB, Stingray Touch after human contact. [file Presentation_4.PDF]
