# Supplementary material for: Aquarium Viromes: Viromes of Human-Managed Aquatic Systems
Source: Front Microbiol. 2017 Jun 30;8:1231. doi: 10.3389/fmicb.2017.01231 (PMC5492393; doi:10.3389/fmicb.2017.01231)
Supplement: Supplementary file 5 [file Table_1.PDF]

Table S1. Characteristics of aquarium exhibits

Water type	Exhibit (Abbreviation)	Water volume (gallon)	Number of animal types	Total number of individual animals	Animal density[a]
Freshwater	Colder Great Lakes (GLB)	1,200	1	45	0.03750
	Warmer Great Lakes (GLA)	44,200	145	759	0.01717
	Amazon Rising (AZ)	16,500	27	64	0.00388
Seawater	Oceanarium (OC)	3,000,000	3	16	0.00001
	Wild Reef (WR)	403,000	49	134	0.00033
	Caribbean Reef (CR)	90,000	102	958	0.01064
	Stingray Touch before human contact (STA)	18,000	0	0	0
	Stingray Touch after human contact (STB)	18,000	7	42	0.00233

[a] Total number of individual animals divided by total volumes of water (gallons) in a system.
