# Supplementary material for: Aquarium Viromes: Viromes of Human-Managed Aquatic Systems
Source: Front Microbiol. 2017 Jun 30;8:1231. doi: 10.3389/fmicb.2017.01231 (PMC5492393; doi:10.3389/fmicb.2017.01231)
Supplement: Supplementary file 6 [file Table_2.PDF]

Table S2. Summary of the sequence reads and the assembled contigs of the aquarium viromes

Aquarium samples	# Raw reads	# Trimmed reads	# Contigs	Mean contig length (bp)	Max contig length (bp)	Min contig length (bp)	Mapping rate (%)[a]	# Contigs assigned[b]	# Contigs to *16S rRNA* genes
AZ-1	38,551,320	38,350,705	53,729	813	62,020	202	83.57	13,676 (25.5%)	0
AZ-2	35,535,018	35,421,663	53,777	918	92,376	200	85.12	14,427 (26.8%)	0
CR-1	75,997,268	75,398,428	113,294	996	167,956	200	88.81	27,509 (24.3%)	0
CR-2	30,566,008	30,450,531	67,101	947	103,146	202	84.36	15,859 (23.6%)	0
GLA-1	49,849,570	49,531,677	56,466	895	236,515	200	86.66	13,754 (24.4%)	0
GLA-2	34,612,536	34,513,506	46,348	871	251,494	200	88.93	10,970 (23.7%)	0
GLB-1	44,830,584	44,594,406	12,080	881	40,008	203	81.86	2,936 (24.3%)	0
GLB -2	23,127,664	23,059,335	9,868	869	48,755	202	71.23	2,331 (23.6%)	0
OC-1	41,380,744	41,171,371	27,338	955	135,498	205	82.14	6,813 (24.9%)	0
OC-2	16,868,024	16,814,171	16,471	858	168,761	216	79.28	3,972 (24.1%)	0
STA-1	53,811,186	53,489,202	12,001	1,033	114,052	200	80.67	3,138 (26.1%)	0
STA-2	34,254,612	34,171,204	8,354	1,015	94,547	200	83.55	2,186 (26.2%)	0
STB-1	134,986,068	133,206,912	91,743	1,011	78,605	200	73.39	23,348 (25.4%)	3 (0.13‰)
STB-2	144,971,864	143,113,052	110,147	1,031	89,267	200	80.71	28,864 (26.2%)	2 (0.07‰)
STB-3	102,958,250	101,726,853	74,452	1,012	81,373	200	82.47	19,414 (26.1%)	0
WR-1	32,872,910	32,649,858	83,437	973	106,273	204	88.39	21,405 (25.7%)	1 (0.05‰)
WR-2	40,186,058	40,016,652	91,580	872	177,566	200	85.63	21,740 (23.7%)	0
**Grand total**	**935,359,684**	**927,679,526**	**928,186**					**232,342**	

[a] Percentage of mapped reads on assembled contigs.

[b] Number of contigs with significant BLASTX alignments (E value < $1.0E^{-5}$) in the NCBI viral reference sequence database.

Abbreviations: AZ, Amazon Rising; CR, Caribbean Reef; GLA, Warmer Great Lakes; GLB, Colder Great Lakes; OC, Oceanarium; STA, Stingray Touch before human contact; STB, Stingray Touch after human contact; WR; Wild Reef.
