# Supplementary material for: Aquarium Viromes: Viromes of Human-Managed Aquatic Systems
Source: Front Microbiol. 2017 Jun 30;8:1231. doi: 10.3389/fmicb.2017.01231 (PMC5492393; doi:10.3389/fmicb.2017.01231)
Supplement: Supplementary file 7 [file Table_3.PDF]

Table S3. Richness estimates for aquarium viromes using CatchAll

Aquarium samples	Best model	Estimated species	Standard error
AZ-1	ThreeMixedExp	88,237.0	22,332.4
AZ-2	ThreeMixedExp	111,532.7	24,998.3
CR-1	ThreeMixedExp	266,169.0	101,797.3
CR-2	ThreeMixedExp	255,191.1	101,797.3
GLA-1	ThreeMixedExp	113,509.7	25,352.8
GLA-2	ThreeMixedExp	89,751.8	18,564.0
GLB-1	ThreeMixedExp	31356.9	10900.3
GLB -2	ThreeMixedExp	30671.4	9304.3
OC-1	ThreeMixedExp	60,641.8	11,762.1
OC-2	ThreeMixedExp	40,066.5	7,688.8
STA-1	ThreeMixedExp	34,733.1	8,036.8
STA-2	ThreeMixedExp	20,121.0	4,423.6
STB-1	ThreeMixedExp	159,378.0	40,020.4
STB-2	ThreeMixedExp	197,364.2	60,923.0
STB-3	ThreeMixedExp	141,850.5	28,411.7
WR-1	ThreeMixedExp	211,489.6	47,353.6
WR-2	ThreeMixedExp	165,755.7	33,838.0

Abbreviations: AZ, Amazon Rising; CR, Caribbean Reef; GLA, Warmer Great Lakes; GLB, Colder Great Lakes; OC, Oceanarium; STA, Stingray Touch before human contact; STB, Stingray Touch after human contact; WR; Wild Reef.
